# Unconventional Human T Cells Accumulate at the Site of Infection in Response to Microbial Ligands and Induce Local Tissue Remodeling

**DOI:** 10.4049/jimmunol.1600990

**Published:** 2016-08-15

**Authors:** Anna Rita Liuzzi, Ann Kift-Morgan, Melisa Lopez-Anton, Ida M. Friberg, Jingjing Zhang, Amy C. Brook, Gareth W. Roberts, Kieron L. Donovan, Chantal S. Colmont, Mark A. Toleman, Timothy Bowen, David W. Johnson, Nicholas Topley, Bernhard Moser, Donald J. Fraser, Matthias Eberl

**Affiliations:** * Division of Infection and Immunity, School of Medicine, Cardiff University, Cardiff CF14 4XN, United Kingdom;; † Wales Kidney Research Unit, Heath Park Campus, Cardiff CF14 4XN, United Kingdom;; ‡ Directorate of Nephrology and Transplantation, Cardiff and Vale University Health Board, University Hospital of Wales, Cardiff CF14 4XW, United Kingdom;; § Department of Renal Medicine, University of Queensland at Princess Alexandra Hospital, Brisbane, Queensland 4102, Australia;; ¶ Centre for Kidney Disease Research, Translational Research Institute, Brisbane, Queensland 4102, Australia;; ‖ Australia and New Zealand Dialysis and Transplant Registry, Adelaide, South Australia 5001, Australia;; # Centre for Medical Education, School of Medicine, Cardiff University, Cardiff CF14 4XN, United Kingdom; and; ** Systems Immunity Research Institute, Cardiff University, Cardiff CF14 4XN, United Kingdom

## Abstract

The antimicrobial responsiveness and function of unconventional human T cells are poorly understood, with only limited access to relevant specimens from sites of infection. Peritonitis is a common and serious complication in individuals with end-stage kidney disease receiving peritoneal dialysis. By analyzing local and systemic immune responses in peritoneal dialysis patients presenting with acute bacterial peritonitis and monitoring individuals before and during defined infectious episodes, our data show that Vγ9/Vδ2^+^ γδ T cells and mucosal-associated invariant T cells accumulate at the site of infection with organisms producing (*E*)-4-hydroxy-3-methyl-but-2-enyl pyrophosphate and vitamin B_2_, respectively. Such unconventional human T cells are major producers of IFN-γ and TNF-α in response to these ligands that are shared by many microbial pathogens and affect the cells lining the peritoneal cavity by triggering local inflammation and inducing tissue remodeling with consequences for peritoneal membrane integrity. Our data uncover a crucial role for Vγ9/Vδ2 T cells and mucosal-associated invariant T cells in bacterial infection and suggest that they represent a useful predictive marker for important clinical outcomes, which may inform future stratification and patient management. These findings are likely to be applicable to other acute infections where local activation of unconventional T cells contributes to the antimicrobial inflammatory response.

## Introduction

The classical view of the immune response to infection is based on the assumption that “innate” immune cells sense pathogens via pattern recognition receptors such as the TLRs, whereas Ag-specific “adaptive” T cell responses are restricted by molecules of the MHC. This simplistic model is being eroded by the growing realization that substantial numbers of T cells in the body are non-MHC restricted and integrate innate and adaptive features (1, 2). Such innate-like T cells include γδ T cells, mucosal-associated invariant T (MAIT) cells, NKT cells, germline-encoded mycolyl-reactive T cells and other CD1-restricted T cells (3–8). Together, these “unconventional” T cell populations make up a sizeable proportion of all T cells in blood, epithelia, organs such as the liver, and inflamed tissues. Sensing nonpeptide Ags by unconventional T cells endows the body with the capacity to respond to a plethora of foreign and self-molecules produced by invading pathogens or released by stressed, infected, or metabolically active tissues. In humans, microbial organisms sensed by one or more of these T cell subsets include the causative agents of tuberculosis, malaria, and most hospital-acquired bacterial infections (9).

There is an increasing appreciation of the role played by unconventional T cells in orchestrating early cellular events in response to invading pathogens, which is likely to contribute to microbial clearance and the development of immunological memory, but which may also result in inflammation-associated tissue damage (1, 9–11). Progress in the understanding of unconventional T cell biology has been hampered by their responsiveness to relatively poorly defined structures and the paucity of appropriate experimental research tools. These cells also display striking species-specific differences with respect to their TCR repertoires, activities, and anatomical locations, with the restricting elements butyrophilin-3 (BTN3), CD1a, CD1b, and CD1c as well as the corresponding T cell subpopulations being absent in mice (1, 8, 9, 12). As a consequence, no small animal model replicates the complex interactions between unconventional T cells and other immune and nonimmune cells in the human body.

The characterization of unconventional T cell responses in vivo and their relevance for homeostasis, immune surveillance, and inflammation remains challenging (3–9). In particular, the microbial and environmental signals that lead to the migration, differentiation, expansion, and maintenance of unconventional T cells under physiological conditions are poorly defined. Studies into human responses during acute infections are notoriously difficult to undertake, with only limited access to relevant specimens, in particular from the site of infection, and to matched samples collected before disease onset. In this study, we addressed this knowledge gap by studying a well-defined cohort of individuals receiving peritoneal dialysis (PD) and presenting with acute peritonitis. PD is a live-saving treatment for people with end-stage kidney disease that permits immunological investigations with direct clinical relevance, where a permanently inserted catheter affords continuous and noninvasive access to localized responses to a range of bacterial species (13–15). We recently reported elevated numbers of Vγ9/Vδ2 T cells in a cross-sectional cohort of PD patients with acute peritonitis, particularly in those with infections caused by Gram-negative and (*E*)-4-hydroxy-3-methyl-but-2-enyl pyrophosphate (HMB-PP)–producing bacteria (14, 15). However, it is unclear whether those elevated numbers are due to preferential recruitment of unconventional T cells to the peritoneal cavity in certain infections and/or a result of ligand-specific local activation and expansion in response to the respective pathogens.

Our present data show that both Vγ9/Vδ2 T cells and MAIT cells specifically accumulate at the site of infection in response to organisms producing HMB-PP and vitamin B_2_, respectively, and have the capacity to activate local tissues with consequences for acute inflammation, peritoneal membrane integrity, and clinical outcomes. In a wider context, the current study demonstrates the power of using PD as an experimental and clinical model for monitoring individuals before, during, and after defined microbial infections.

## Materials and Methods

### Study approval

Recruitment of PD patients and healthy volunteers for this study was approved by the South East Wales Local Ethics Committee under reference numbers 04WSE04/27 and 08/WSE04/17, respectively, and conducted according to the principles expressed in the Declaration of Helsinki. All individuals provided written informed consent. The PD study was registered on the U.K. Clinical Research Network Study Portfolio under reference numbers 11838 “Patient immune responses to infection in Peritoneal Dialysis” (PERIT-PD) and 11839 “Leukocyte phenotype and function in Peritoneal Dialysis” (LEUK-PD). Fresh omentum samples from consented patients were obtained from the Wales Kidney Research Tissue Bank.

### Patient samples

The local study cohort comprised 101 adults PD patients admitted to the University Hospital of Wales, Cardiff, on day 1 of acute peritonitis between September 2008 and April 2016. Forty-one stable individuals receiving PD for at least 3 mo and with no previous infection served as noninfected controls. Subjects known to be positive for HIV or hepatitis C virus were excluded. Clinical diagnosis of acute peritonitis was based on the presence of abdominal pain and cloudy peritoneal effluent with >100 WBCs/mm^3^. According to the microbiological analysis of the effluent by the routine Microbiology Laboratory, Public Health Wales, episodes of peritonitis were defined as culture negative (with unclear etiology) or as confirmed bacterial infections caused by specific subgroups of Gram-positive and Gram-negative organisms. The distribution of the nonmevalonate (HMB-PP) and vitamin B_2_ pathways across microbial species was determined based on the absence or presence of the enzymes HMB-PP synthase (EC 1.17.7.1) and 6,7-dimethyl-8-d-ribityllumazine (DMRL) synthase (EC 2.5.1.78), respectively, in the corresponding genomes, according to the Kyoto Encyclopedia of Genes and Genomes (KEGG; http://www.genome.jp/kegg). Cases of fungal infection and mixed or unclear culture results were excluded from this analysis.

### Outcome analysis

Microbiological and clinical outcome data were obtained from 5071 adult patients of the Australia and New Zealand Dialysis Transplant (ANZDATA) Registry who developed first-time peritonitis between April 2003 and December 2012, excluding cases of fungal infection, polymicrobial infection, or unrecorded culture results. Clinical outcomes examined were cessation of therapy because of catheter removal in 90 d, transfer to permanent hemodialysis (HD) in 90 d, transfer to interim HD for at least 30 d and death in 30 d, as well as the individuals outcomes combined to yield the overall technique failure. Outcome predictors were determined using binary logistic regression and forward elimination of data. Survival analyses were performed using the Kaplan–Meier approach, and differences between groups were assessed using log-rank tests.

### Media, reagent, and Abs

Peritoneal leukocytes were cultured in RPMI 1640 medium supplemented with 2 mM l-glutamine, 1% sodium pyruvate, 50 μg/ml penicillin/streptomycin, and 10% FCS (Life Technologies). Mesothelial cells were cultured in Earle’s buffered Medium 199 (Life Technologies) containing 10% FCS, and peritoneal fibroblasts were cultured in a 1:1 (v/v) mixture of DMEM and Ham’s F-12 nutrients (Life Technologies) with 20% FCS; both media were supplemented with 100 U/ml penicillin, 100 μg/ml streptomycin, and 2 mM l-glutamine (Life Technologies) as well as 5 μg/ml transferrin, 5 μg/ml insulin, and 0.4 μg/ml hydrocortisone (all from Sigma-Aldrich). Synthetic (*E*)-4-hydroxy-3-methyl-but-2-enyl pyrophosphate (HMB-PP) was provided by Dr. H. Jomaa (University of Giessen, Giessen, Germany) and synthetic DMRL by Dr. B. Illarionov (Hamburg School of Food Science, Hamburg, Germany). Biotinylated monomers of human MHC-related protein 1 (MR1) loaded with reduced 6-hydroxymethyl-8-(1-D-ribityl) lumazine (active ligand) or 6-formylpterin (negative control) were provided by Dr. L. Kjer-Nielsen (University of Melbourne, Melbourne, VIC, Australia) and reconstituted as described before (16). rIFN-γ, rTNF-α, and rIL-1β were purchased from Miltenyi Biotec. Human T-Activator CD3/CD28 Dynabeads were purchased from Life Technologies. Blocking reagents used included anti-BTN3 (103.2; Dr. D. Olive, Université de la Méditerranée, Marseille, France); anti-MR1 (26.5; Dr. T. Hansen, Washington University School of Medicine, St. Louis, MO); anti–IFN-γ (B27) and anti–IL-1β (H1b-27) (BioLegend); and sTNFR p75-IgG1 fusion protein (etanercept/Enbrel; Amgen).

### Bacteria

Clinical isolates of *Escherichia coli* (Gram^−^HMB-PP^+^vit.B2^+^), *Klebsiella pneumoniae* (Gram^−^HMB-PP^+^vit.B2^+^), *Pseudomonas aeruginosa* (Gram^−^HMB-PP^+^vit.B2^+^), *Corynebacterium striatum* (Gram^+^HMB-PP^+^vit.B2^+^), *Listeria monocytogenes* (Gram^+^HMB-PP^+^vit.B2^−^), *Staphylococcus aureus* (Gram^+^HMB-PP^−^vit.B2^+^), *Streptococcus pneumoniae* (Gram^+^HMB-PP^−^vit.B2^−^), and *Enterococcus faecalis* (Gram^+^HMB-PP^−^vit.B2^−^) were grown in Luria–Bertani broth, harvested at an OD_600_ of 0.5–0.8, and sonicated in 1/10 (v/v) PBS (pH 8). Insoluble debris was removed by centrifugation, the supernatants were passed through 0.1-μm sterile filter units (Millipore), and the protein concentrations were determined using the BCA protein assay kit (Pierce). Low molecular mass fractions were obtained using cellulose filters with a molecular mass cutoff of 3 kDa (Millipore). Bacterial extracts were used in cell culture at dilutions corresponding to protein concentrations of the original samples (before 3-kDa filtration) of 60–100 μg/ml.

### T cells

PBMC were isolated from peripheral blood of healthy volunteers using Lymphoprep (Axis-Shield). Vγ9^+^ T cells (>98%) were isolated from PBMC using mAbs against Vγ9-PECy5 (Beckman Coulter) and anti-PE magnetic microbeads (Miltenyi Biotec); Vα7.2^+^ T cells (>98%) were isolated using anti–Vα7.2-allophycocyanin (BioLegend) and anti-allophycocyanin microbeads (Miltenyi Biotec). To generate unconventional T cell–conditioned medium, purified blood Vγ9/Vδ2 T cells and MAIT cells were incubated for 24 h in the presence of 10 nM HMB-PP and anti-CD3/CD28 Dynabeads at 0.5 beads/cell, respectively. Human peritoneal leukocytes were harvested from overnight dwell effluents of stable PD patients (13) and cultured in the absence or presence of 1–100 nM HMB-PP, 100 μM DMRL, or bacterial extracts at dilutions corresponding to protein concentrations of 60–100 μg/ml. For blocking experiments, anti-BTN3 and anti-MR1 were used at 10 μg/ml and added 30 min before stimulating the cells.

### Mesothelial cells and peritoneal fibroblasts

Human peritoneal mesothelial cells were obtained from fresh omental samples after two cycles of tissue digestion in the presence of trypsin (15 min each); peritoneal fibroblasts were obtained after a third digestion cycle lasting 1 h (17–19). All data presented are from experiments performed with confluent mesothelial cells and fibroblasts between the first and third passage. Mesothelial cells were growth arrested for 48 h in serum-free medium prior to treatment; fibroblasts were growth arrested in medium containing 0.2% FCS. After starvation, cells were exposed for 24 h to T cell–conditioned medium at the indicated dilutions; rTNF-α and rIFN-γ were used as controls. Cell-free peritoneal effluent from stable and infected patients (*n* = 3–4) was added to cell cultures at a dilution of 1:4. In blocking experiments, T cell–conditioned medium or peritoneal effluent were pretreated for 30 min with anti–IFN-γ, anti–IL-1β, and sTNFR, either alone or in combination at 10 μg/ml. Supernatants were harvested and assessed by ELISA; cells were analyzed by quantitative PCR.

### Flow cytometry

Cells were acquired on an eight-color FACSCanto II (BD Biosciences) and analyzed with FlowJo 10.1 (Tree Star), using mAbs against CD3 (SK7), CD69 (FN50), CCR4 (1G1), CCR5 (2D7), and CCR6 (11A9) from BD Biosciences; anti–TCR-Vγ9 (Immu360) from Beckman Coulter; and anti-CD161 (HP-3G10), CCR2 (K036C2), anti–TCR-Vα7.2 (3C10) (BioLegend), together with appropriate isotype controls. Anti-mouse beads were used to set compensation (Life Technologies). Intracellular cytokines were detected using anti–IFN-γ (B27; BioLegend) and anti–TNF-α (188; Beckman Coulter). For detection of intracellular cytokines, 10 μg/ml brefeldin A (Sigma-Aldrich) was added to cultures 5 h prior to harvesting. Leukocyte populations were gated based on their appearance in side scatter and forward scatter area/height and exclusion of live/dead staining (fixable Aqua; Invitrogen). Unless stated otherwise, peritoneal γδ T cells were defined as Vγ9^+^CD3^+^ lymphocytes. Peritoneal MAIT cells were defined as Vα7.2^+^CD161^+^CD3^+^ lymphocytes; control stainings using MR1 tetramers as reference confirmed the validity of this approach (data not shown).

### ELISA

Cell-free peritoneal effluents were analyzed on a SECTOR Imager 6000 (Meso Scale Discovery) for IFN-γ, TNF-α, IL-1β, CCL3, CCL4, and CXCL8. Conventional ELISA kits and a Dynex MRX II reader were used for CCL2 (eBioscience) and CCL20 (R&D Systems). Cell culture supernatants were analyzed using conventional ELISA kits for IFN-γ (BioLegend), TNF-α and CCL2 (eBioscience) as well as for CXCL8, CXCL10, and IL-6 (R&D Systems).

### Real-time PCR

Total RNA was isolated from mesothelial cells cultured under the indicated conditions using TRIzol (Invitrogen). cDNA was generated from 0.5 μg of RNA using the high-capacity cDNA reverse transcription kit (Thermo Fisher), 100 mM 2′-deoxynucleoside 5′-triphosphates, 40 U/μl RNase inhibitor (New England Biolabs), 50 U/μl MultiScribe reverse transcriptase, and 1× random primers, according to the manufacturer’s recommendations. Quantitative PCRs were run on a ViiA7 real-time PCR system (Thermo Fisher), using the power SYBR green PCR master mix (Thermo Fisher) and 300 nM forward and reverse primers: 5′-TCCCAATACATCTCCCTTCACA-3′ and 5′-ACCCACCTCTAAGGCCATCTTT-3′ for E-cadherin; 5′-TAAATCCACGCCGGTTCCTGAAGT-3′ and 5′-AGGTGTCTCAAAGTTACCACCGCT-3′ for occludin; 5′-CCGAGGTTTTAACTGCGAGA-3′ and 5′-TCACCCACTCGGTAAGTGTTC-3′ for fibronectin; 5′-CGAGCCCACCGGGAACGAAA-3′ and 5′-GGACCGAAGGCGCTTGTGGAG-3′ for IL-6; 5′-CCTCTGACTTCAACAGCGACAC-3′ and 5′-TGTCATACCAGGAAATGAGCTTGA-3′ for GAPDH; 5′-TTTACCTTCCAGCAGCCCTA-3′ and 5′-GGACAGAGTCCCAGATGAGC-3′ for Snail; 5′-AACTGGGACGACATGGAAA-3′ and 5′-AGGGTGGGATGCTCTTCAG-3′ for α-smooth muscle actin; 5′-GGAGAGGTGTTCCGTGTTGT-3′ and 5′-GGCTAGCTGCTCAGCTCTGT-3′ for zona occludens-1; and 5′-CGGGTTGCTTGCAATGTGC-3′ and 5′-CCGGCGACAACATCGTGAC-3′ for claudin-1. Ten nanograms of total RNA were used for cellular microRNAs (miRs) using the TaqMan Universal Master Mix II and specific primers for miR-21 and miR-191 (Applied Biosystems). mRNA and miR expression levels were normalized to the endogenous controls GAPDH and miR-191, respectively.

### Statistics

Statistical analyses were performed using GraphPad Prism 6.0 software. Data distributions were analyzed using D’Agostino–Pearson omnibus normality tests. Data were analyzed using two-tailed Student *t* tests for normally distributed data and two-tailed Mann–Whitney tests for non-parametric data. Differences between groups were analyzed using one-way ANOVA with Holm–Sidak’s post tests for multiple comparisons of parametric data, or Kruskal–Wallis tests combined with Dunn’s post tests for nonparametric data. Matched data were analyzed using paired *t* tests or Wilcoxon matched-pairs tests for two groups, or Friedman tests combined with Dunn’s multiple comparisons tests for more than two groups. Differences were considered statistically significant as indicated in the figures and tables: *p < 0.05, **p < 0.01, and ***p < 0.001.

## Results

### Blood unconventional T cells migrate early into the inflamed peritoneal cavity, at the time of peak neutrophil influx

**FIGURE 1.**
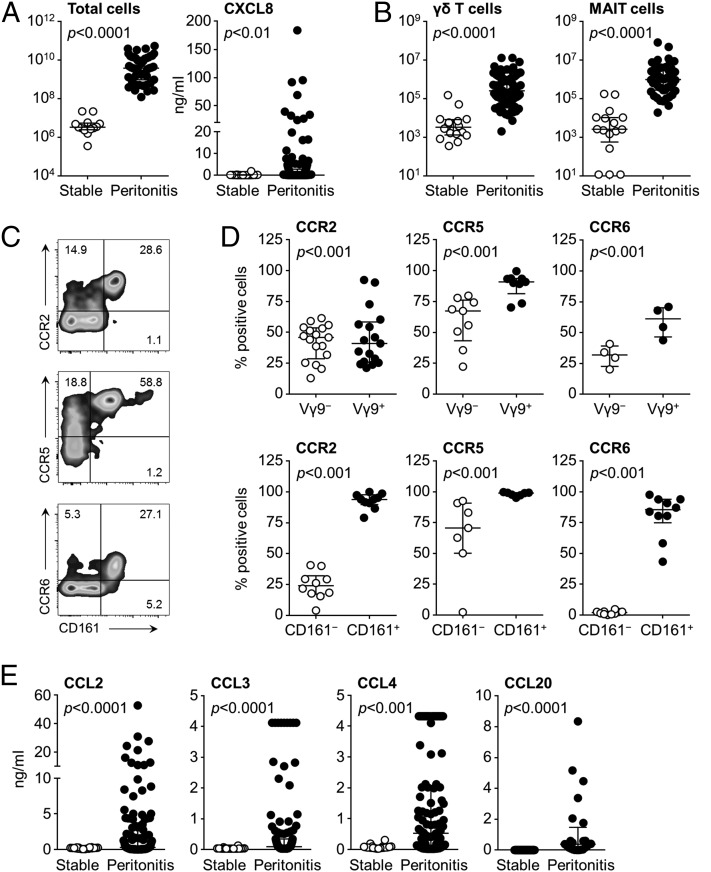
Proinflammatory migratory profile of unconventional T cells. (**A**) Total cell counts and concentration of the neutrophil-attracting chemokine CXCL8 in the peritoneal effluent of stable PD patients and patients presenting with acute peritonitis. (**B**) Total numbers of Vγ9^+^CD3^+^ T cells and Vα7.2^+^CD3^+^ T cells within the peritoneal cell population in stable PD patients and during acute peritonitis. (**C**) Representative example for the coexpression of CCR2, CCR5, and CCR6 with CD161 on blood Vα7.2^+^CD3^+^ T cells in a stable PD patient. (**D**) Percentage of CCR2^+^, CCR5^+^, and CCR6^+^ cells among Vγ9^−^ and Vγ9^+^CD3^+^ T cells (upper panels) or among Vα7.2^+^CD161^−^ and Vα7.2^+^CD161^+^CD3^+^ T cells in the blood of stable PD patients (lower panels). (**E**) Concentration of the indicated chemokines in the effluent of patients presenting with acute peritonitis; upper limits of detection for CCL3 and CCL4 were 4.12 and for 4.32 ng/ml, respectively. Data were analyzed using Mann–Whitney tests (in the case of CCL2 after normalization). Each data point represents an individual patient, error bars depict the median ± interquartile range.

**FIGURE 2.**
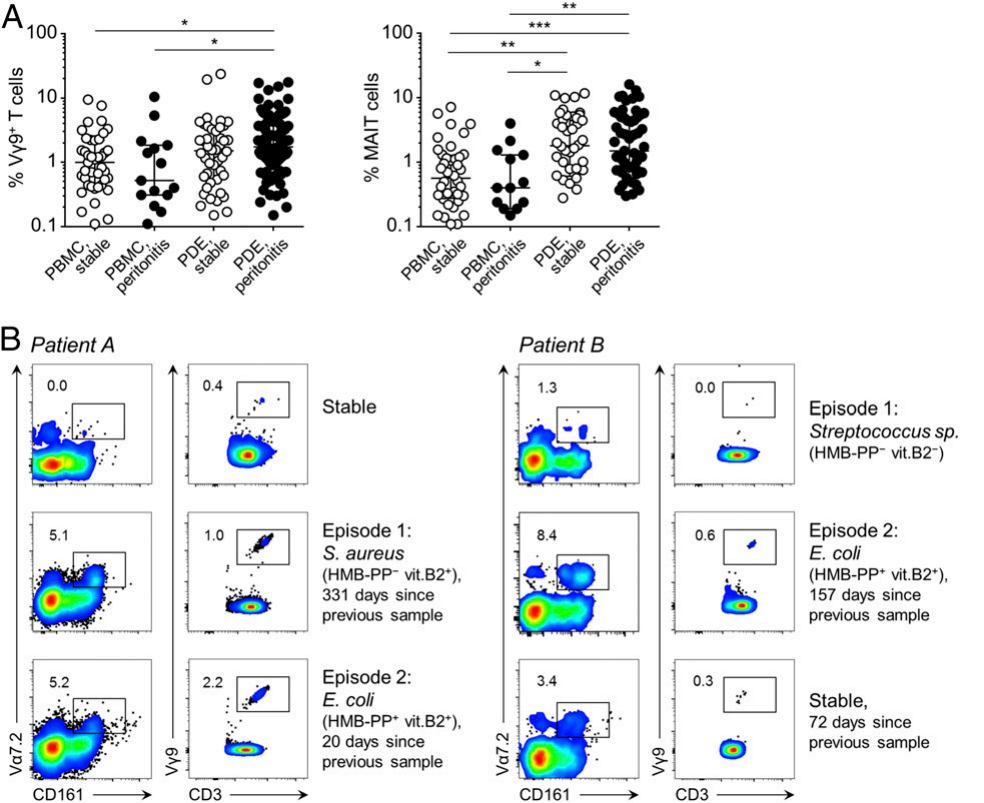
Systemic and local levels of unconventional T cells in stable PD patients and during acute peritonitis. (**A**) Blood (PBMC) and peritoneal dialysis effluent (PDE) were analyzed by flow cytometry for the proportion of Vγ9/Vδ2 T cells (identified as Vγ9^+^; left) and MAIT cells (Vα7.2^+^CD161^+^; right), expressed as percentage of all CD3^+^ T cells. Samples were collected from stable PD patients and patients presenting with acute peritonitis (day 1), before commencing antibiotic treatment. Data were analyzed using Kruskal–Wallis tests combined with Dunn’s multiple comparisons tests. Each data point represents an individual patient; asterisks indicate significant differences between groups. (**B**) Local levels of unconventional T cells in the effluent of two PD patients while the individuals were stable and when they presented with distinct peritonitis episodes caused by bacteria capable or not of producing HMB-PP or vitamin B2 (day 1).

Acute disease is characterized by a considerable influx of leukocytes to the site of infection, together with locally elevated levels of inflammatory mediators. In this study, the effluent of PD patients presenting with acute peritonitis, before commencing antibiotic treatment, contained increased levels of total cells and of chemokines like the neutrophil-attracting molecule CXCL8 (IL-8), compared with effluent from stable noninfected PD patients (Fig. 1A). Absolute numbers of both Vγ9/Vδ2 T cells and MAIT cells were increased during acute peritonitis compared with stable patients (Fig. 1B), indicating rapid corecruitment of unconventional T cells from blood to the inflamed peritoneal cavity, alongside neutrophils. These observations were in agreement with the migratory profiles of these cells. Circulating Vγ9/Vδ2 T cells and MAIT cells in stable PD patients preferentially expressed the chemokine receptors CCR2, CCR5, and CCR6, compared with their non-Vγ9 and non-MAIT counterparts (Fig. 1C, 1D). We detected markedly increased levels of the corresponding chemokines CCL2 (CCR2 ligand), CCL3 and CCL4 (CCR5 ligands), and CCL20 (CCR6 ligand) in early peritonitis (Fig. 1E), demonstrating a substantial production of inflammatory chemokines with the potential to attract unconventional T cells to the site of infection, thereby complementing the local pool of tissue-resident Vγ9/Vδ2 T cells and MAIT cells. In accordance, local frequencies of Vγ9/Vδ2 T cells and MAIT cells during peritonitis were generally higher than in blood (Fig. 2A). No systemic increase in blood unconventional T cell levels was seen in patients with peritonitis, in support of the locally confined nature of the infection.

### Unconventional T cells are locally enriched during acute infections caused by bacterial pathogens producing the corresponding ligands

To avoid confounding resulting from the considerable biological variations between people and the underlying pathologies, we collected matched samples from the same individuals to examine systemic responses in blood and local responses in the peritoneal cavity, before and during episodes of peritonitis. Such investigations in acutely infected people have not been attempted before and are a unique advantage of studying individuals receiving PD. These matched analyses in fact identified further ligand-specific effects on local frequencies because Vγ9/Vδ2 T cells and MAIT cells were particularly elevated in peritonitis caused by bacteria producing HMB-PP and vitamin B_2_, respectively, even in individuals with multiple infection episodes (Fig. 2B).

**FIGURE 3.**
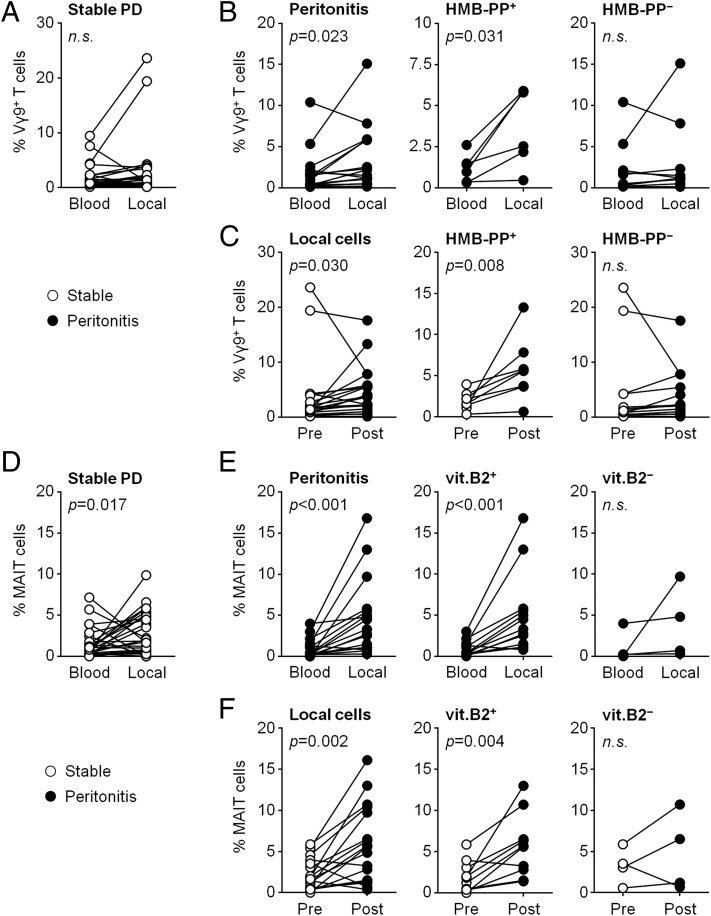
Matched levels of unconventional T cells in blood and effluent of PD patients before and during acute peritonitis. Blood and peritoneal effluent samples from the same individuals were analyzed by flow cytometry for the proportion of Vγ9/Vδ2 T cells (identified as Vγ9^+^) (**A***–***C**) and MAIT cells (Vα7.2^+^ CD161^+^) (**D***–***F**), expressed as percentage of all CD3^+^ T cells. Samples were collected while patients were stable and when they presented with acute peritonitis (day 1), before commencing antibiotic treatment. (A and D) Unconventional T cell levels in blood and effluent of stable individuals. (B and E) Unconventional T cell levels in blood and effluent of all patients with acute peritonitis (left) and in subgroups of patients with confirmed infections by bacteria capable or not of producing HMB-PP or vitamin B_2_ (middle, right). (C and F) Local unconventional T cell levels in the effluent of PD patients before and during acute peritonitis (left) and in subgroups of patients with infections by bacteria producing HMB-PP and/or vitamin B_2_ (middle, right). Data were analyzed using Wilcoxon matched–pairs signed rank tests. Each data point represents an individual patient.

Across the cohort, proportions of Vγ9/Vδ2 T cells among all CD3^+^ T cells in blood and peritoneal cavity were comparable in the absence of infection, indicating that under homeostatic conditions Vγ9/Vδ2 T cells are not enriched locally (Fig. 3A). In contrast, on the day of presentation with acute peritonitis, local levels of Vγ9/Vδ2 T cells were elevated compared with blood, suggesting preferential recruitment and/or accumulation at the site of infection (Fig. 3B). This preferential increase in local Vγ9/Vδ2 T cell levels was apparent in patients infected with HMB-PP^+^ bacteria but not in patients with HMB-PP^−^ infections (Fig. 3B), despite highly elevated peritoneal chemokine levels in both HMB-PP^+^ and HMB-PP^−^ infections (data not shown). Finally, we performed a longitudinal study and observed a significant increase in local Vγ9/Vδ2 T cell levels in patients who developed acute peritonitis caused by HMB-PP^+^ organisms over preinfection baseline levels when the same individuals were stable (Fig. 3C). Because there was no such difference between preinfection and postinfection levels in individuals presenting with HMB-PP^−^ infections, these findings show that Vγ9/Vδ2 T cells accumulate locally at the site of infection in response to HMB-PP^+^ but not HMB-PP^−^ organisms.

Parallel studies on the distribution of MAIT cells revealed analogous patterns. Although in stable PD patients local MAIT cell frequencies in the peritoneal cavity were slightly higher than those in blood (Fig. 3D), such differences between anatomical sites were much more pronounced in acutely infected individuals (average 4.65% of all T cells in the peritoneal cavity versus 0.91% in blood), particularly during infections caused by vitamin B_2_–producing (vit.B2^+^) bacteria (Fig. 3E). As observed for peritoneal Vγ9/Vδ2 T cell responses to HMB-PP^+^ bacteria, a comparison with matched preinfection values revealed a significant local increase in MAIT cell levels in individuals infected with vit.B2^+^ organisms (Fig. 3F), confirming the responsiveness of MAIT cells to vitamin B_2_ derivatives in vivo. These findings on Vγ9/Vδ2 T-cells and MAIT cells support a ligand-induced local expansion of unconventional T-cells at the site of infection, in addition to the ligand-independent recruitment from the blood (Fig. 1B).

### Peritoneal unconventional T cells respond to bacterial pathogens producing the corresponding ligands

**FIGURE 4.**
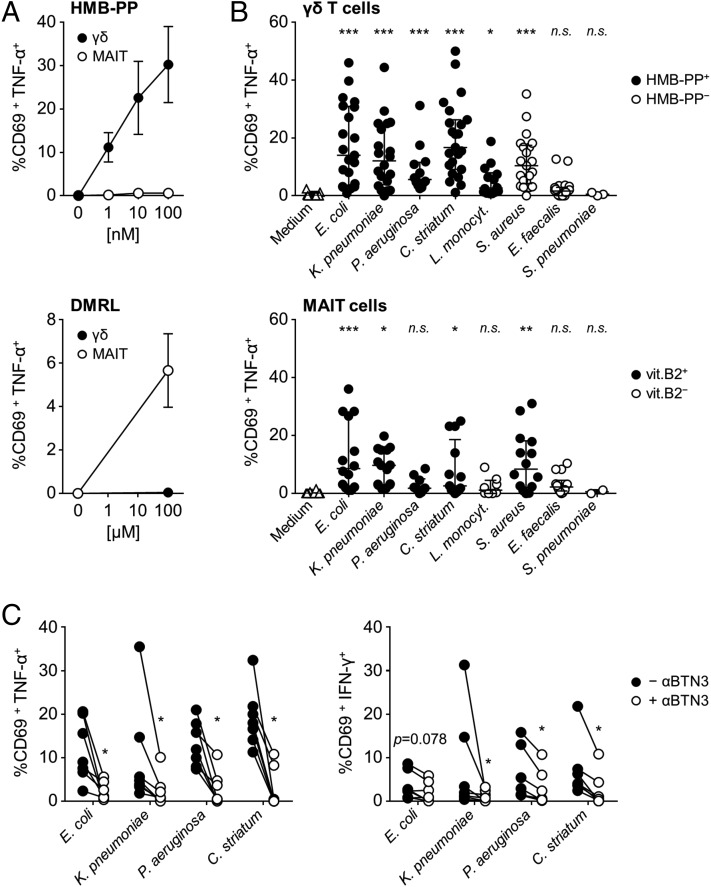
Peritoneal unconventional T cell responses to microbial metabolites. (**A**) Activation of peritoneal Vγ9^+^ γδ T cells and Vα7.2^+^CD161^+^ MAIT cells from stable PD patients upon overnight stimulation with HMB-PP (*n* = 4 individual patients) or DMRL (*n* = 3), as analyzed by flow cytometry and expressed as proportion of γδ or MAIT cells coexpressing CD69 and TNF-α (means ± SEM). (**B**) Activation of peritoneal Vγ9^+^ γδ T cells and Vα7.2^+^CD161^+^ MAIT cells upon overnight stimulation in the presence of extracts from different clinical isolates that produce (filled symbols) or do not produce (empty symbols) the corresponding ligands (median ± interquartile range): *E. coli* (HMB-PP^+^vit.B2^+^), *K. pneumoniae* (HMB-PP^+^vit.B2^+^), *P. aeruginosa* (HMB-PP^+^vit.B2^+^), *C. striatum* (HMB-PP^+^vit.B2^+^), *L. monocytogenes* (HMB-PP^+^ vit.B2^−^), *S. aureus* (HMB-PP^−^vit.B2^+^), *E. faecalis* (HMB-PP^−^vit.B2^−^), and *S. pneumoniae* (HMB-PP^−^vit.B2^−^). Data were analyzed using Kruskal–Wallis tests combined with Dunn’s multiple comparisons tests. Each data point represents an individual patient. (**C**) Activation of total peritoneal leukocytes by extracts of the indicated bacteria, in the absence or presence of anti-BTN3 blocking Abs, shown as coexpression of CD69 and TNF-α (left) or IFN-γ (right) by Vγ9^+^ T cells after overnight stimulation. Data were analyzed using Wilcoxon matched–pairs signed rank tests. Each data point represents an individual patient; asterisks depict significant differences of anti-BTN3–treated samples compared with untreated controls.

To address the pathogen specificity of local unconventional T cell responses, we cultured peritoneal leukocytes from stable PD patients in the presence of different microbial stimuli. In agreement with our earlier data on blood cells (10), peritoneal Vγ9/Vδ2 T cells were highly specific for HMB-PP, whereas MAIT cells recognized the vitamin B_2_ precursor, DMRL, as judged by upregulation of CD69 and production of TNF-α by responding cells (Fig. 4A). When testing extracts from defined clinical isolates covering the majority of pathogens associated with PD-related peritonitis, peritoneal Vγ9/Vδ2 T cells responded readily to HMB-PP–producing Gram-negative (*E. coli*, *K. pneumoniae*, and *P. aeruginosa*) and Gram-positive bacteria (*C. striatum* and *L. monocytogenes*) but not to HMB-PP–deficient *E. faecalis* and *S. pneumoniae* (Fig. 4B). Blocking experiments using neutralizing Abs confirmed that these HMB-PP–dependent responses were mediated via BTN3 (Fig. 4C). Strikingly, peritoneal Vγ9/Vδ2 T cells also responded to *S. aureus* despite this organism’s lack of HMB-PP, possibly via superantigens (20). Peritoneal MAIT cells were activated by the vit.B2^+^ bacteria *E. coli*, *K. pneumoniae*, *C. striatum*, and *S. aureus* but not by the vit.B2^−^ species *L. monocytogenes*, *E. faecalis*, and *S. pneumoniae*; responses to the vit.B2^+^ organism *P. aeruginosa* did not reach statistical significance (Fig. 4B).

**FIGURE 5.**
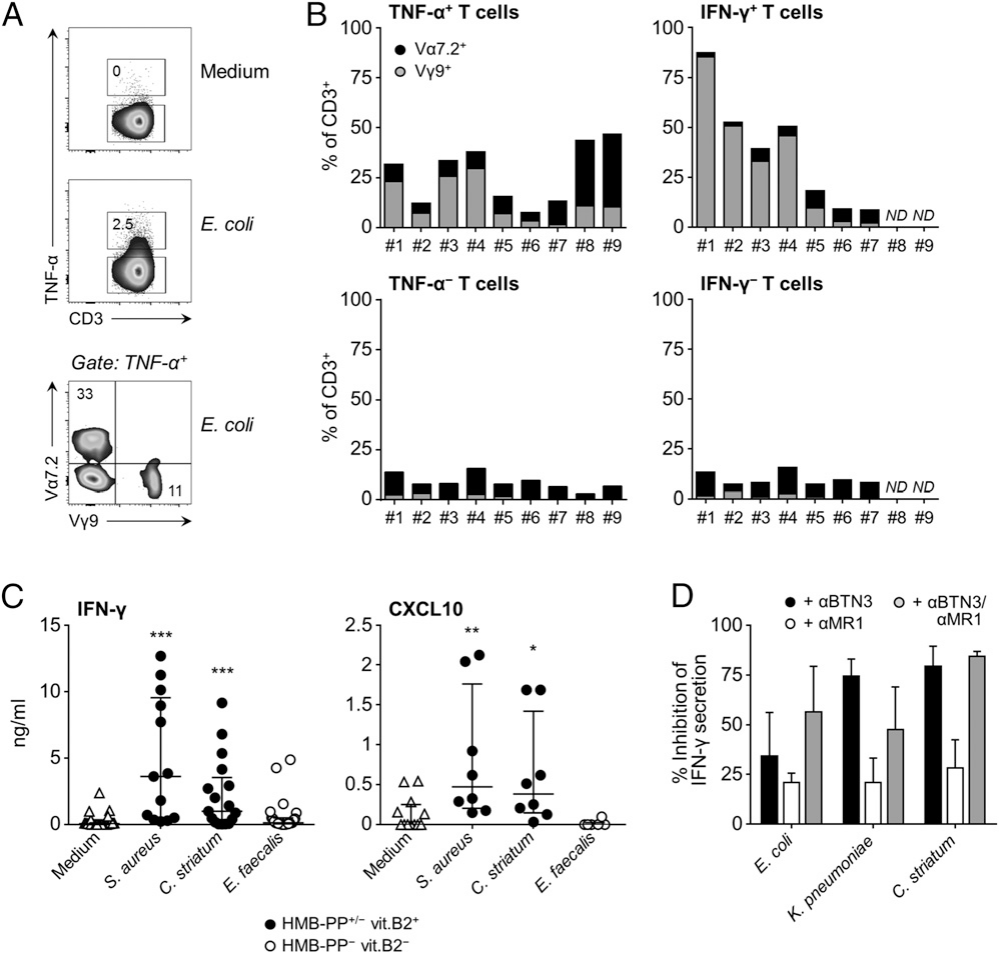
Ex vivo responsiveness of peritoneal leukocytes to pathogenic bacteria. Peritoneal cells were obtained from the effluent of stable patients and exposed overnight to extracts prepared from the indicated bacterial species. (**A**) Representative example of an intracellular staining of TNF-α in peritoneal leukocytes cultured in the absence (medium; top panel) or presence of *E. coli* extract (middle panel), as analyzed by flow cytometry within the CD3^+^ gate. Bottom panel, distribution of Vα7.2^+^ and Vγ9^+^ cells within all CD3^+^TNF-α^+^ peritoneal cells after stimulation with *E. coli* extract. (**B**) Proportion of Vα7.2^+^ (black) and Vγ9^+^ cells (shaded) T cells among peritoneal T cells producing or not TNF-α and IFN-γ in response to *E. coli*, as analyzed by flow cytometry in nine stable individuals. (**C**) Overnight secretion of IFN-γ, CXCL10, and CXCL8 by peritoneal cells in response to bacteria that produce (*S. aureus*, *C. striatum*; filled circles) or do not produce (*E. faecalis*; empty circles) ligands for Vγ9/Vδ2 T cells and/or MAIT cells, as analyzed by ELISA (median ± interquartile range). Data were analyzed using Kruskal–Wallis tests combined with Dunn’s multiple comparisons tests. Each data point represents an individual patient; asterisks indicate significant differences compared with medium controls (triangles). (**D**) Specific inhibition of IFN-γ secretion by peritoneal leukocytes in response to bacterial extracts, in the absence or presence of anti-BTN3 and anti-MR1 blocking Abs, alone or in combination. Data shown are means ± SEM from independent experiments with three omental donors. *ND*, not done.

Although Vγ9/Vδ2 T cells and MAIT cells constitute only minor proportions of the peritoneal T cell pool, these cells represented major producers of proinflammatory cytokines in response to bacterial extracts (Fig. 5A). Using *E. coli* as example of an organism producing both HMB-PP and vitamin B_2_, responding peritoneal Vγ9/Vδ2 T cells and MAIT cells together made up a large fraction of TNF-α^+^ T cells (median 31.7%) and IFN-γ^+^ T cells (median 39.2%) despite considerable variability across PD patients (Fig. 5B). In contrast, both cell types constituted far lower proportions among TNF-α^−^ and IFN-γ^−^ T cells in *E. coli*–stimulated peritoneal leukocytes. Similar results were obtained using HMB-PP^+^vit.B2^+^ *C. striatum* extracts (data not shown). Analyses of supernatants from peritoneal leukocytes exposed to different bacteria demonstrated that only organisms producing HMB-PP and/or vitamin B_2_ (*S. aureus* and *C. striatum*) but not HMB-PP^−^vit.B2^−^ *E. faecalis* induced secretion of IFN-γ and the IFN-γ–inducible chemokine CXCL10 (Fig. 5C). As control, levels of CXCL8 were comparable in response to all three bacterial species, demonstrating an equal potential to stimulate peritoneal leukocytes other than Vγ9/Vδ2 T cells and MAIT cells (data not shown). Secretion of TNF-α was not assessed in these experiments as any unconventional T cell–derived TNF-α would have been masked by peritoneal macrophages and neutrophils sensing diverse pathogen-associated molecular patterns. The contribution of Vγ9/Vδ2 T cells and MAIT cells to the overall secretion of IFN-γ by peritoneal leukocytes in response to HMB-PP^+^vit.B2^+^ organisms was confirmed by anti-BTN3 and anti-MR1 blocking Abs (Fig. 5D). Taken together, these findings show that peritoneal unconventional T cells are major producers of IFN-γ and TNF-α in response to a wide range of microbial pathogens and that inhibition of ligand recognition through BTN3 and MR1 abrogates this cytokine production.

### Cross-talk with local tissue amplifies the proinflammatory response to bacterial pathogens

**FIGURE 6.**
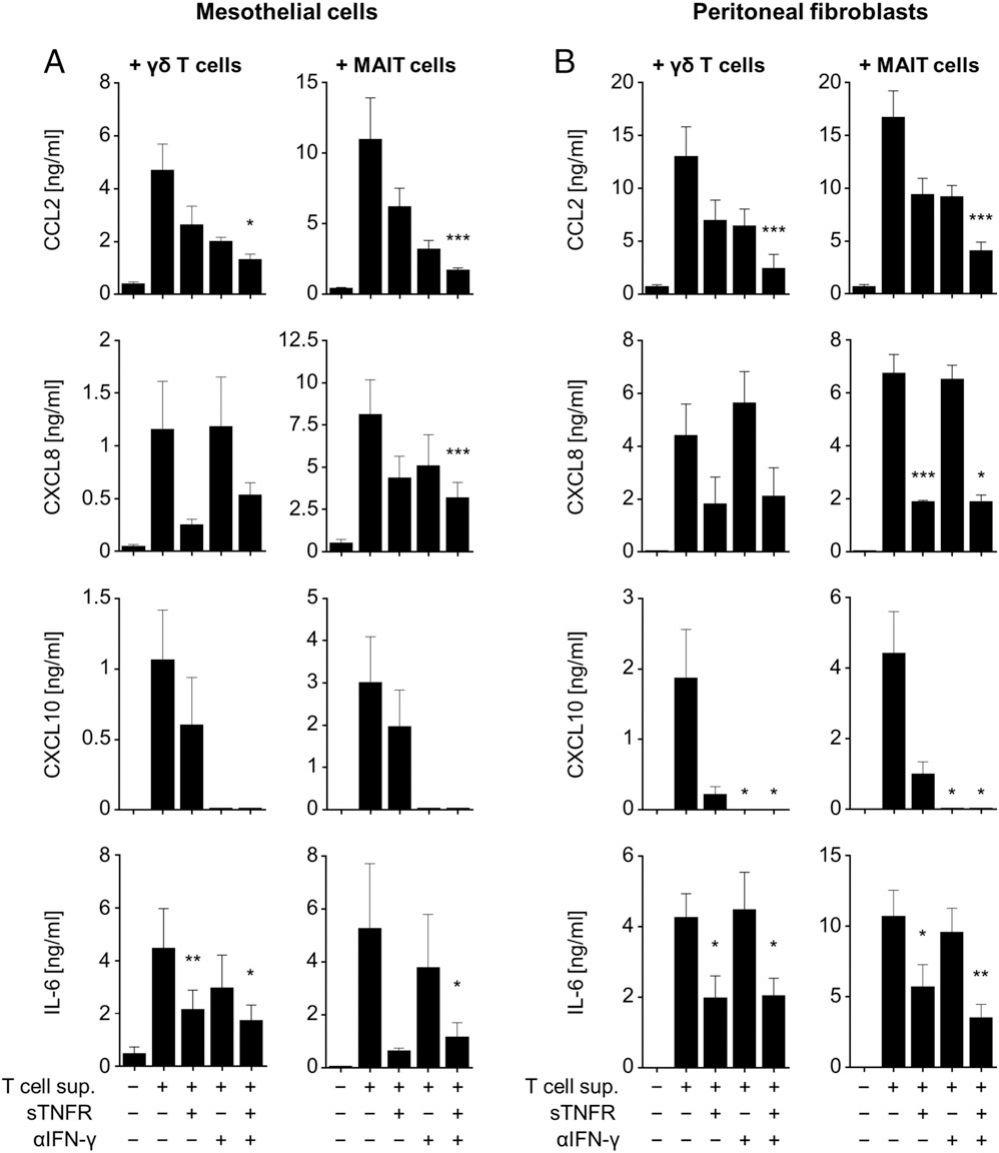
Activation of peritoneal tissue cells by γδ T cell– and MAIT cell–derived cytokines. Growth-arrested peritoneal mesothelial cells (**A**) or peritoneal fibroblasts (**B**) from human omentum were exposed to supernatants derived from activated Vγ9/Vδ2 T cells and MAIT cells at a dilution of 1:4, in the absence or presence of 10 μg/ml sTNFR and 10 μg/ml anti–IFN-γ, alone or together. Data shown are levels of CCL2, CXCL8, CXCL10, and IL-6 secreted into the culture medium over 24 h by ELISA (means ± SEM from independent experiments with four to seven omental donors). Data were analyzed using Friedman tests combined with Dunn’s multiple comparisons tests. Asterisks indicate significant differences compared with medium controls.

Local activation of unconventional T cells in acute infection is likely to occur in close proximity to the peritoneal membrane, thus exposing the mesothelial cell layer that lines the peritoneal cavity to T cell–derived mediators. Using supernatants from activated unconventional T cells, our experiments demonstrate that Vγ9/Vδ2 T cells and MAIT cells induced secretion of CCL2, CXCL8, CXCL10, and IL-6 by omentum-derived primary mesothelial cells (Fig. 6A) and peritoneal fibroblasts (Fig. 6B). This activation of peritoneal tissue cells was dose dependent (data not shown). Neutralization of TNF-α and/or IFN-γ in the conditioned media prior to addition to peritoneal tissue cells attenuated these responses considerably, with the CXCL8 and IL-6 secretion being particularly sensitive to inhibition of TNF-α, whereas the CXCL10 secretion was mainly driven by IFN-γ (Fig. 6). These findings were in accordance with control experiments showing that rTNF-α was a potent inducer of CCL2, CXCL8, and IL-6 expression by mesothelial cells and fibroblasts (data not shown). rIFN-γ on its own was mainly effective at inducing CCL2 and, to a lesser extent, IL-6 expression by mesothelial cells and fibroblasts while having no effect on CXCL8. These findings identify unconventional T cell–derived TNF-α and IFN-γ as major stimulators of peritoneal tissue cells, which is likely to enhance local inflammation and contribute to further recruitment of monocytes, neutrophils and lymphocytes to the site of infection.

### Activation of peritoneal tissue cells by effluent from PD patients with acute peritonitis

**FIGURE 7.**
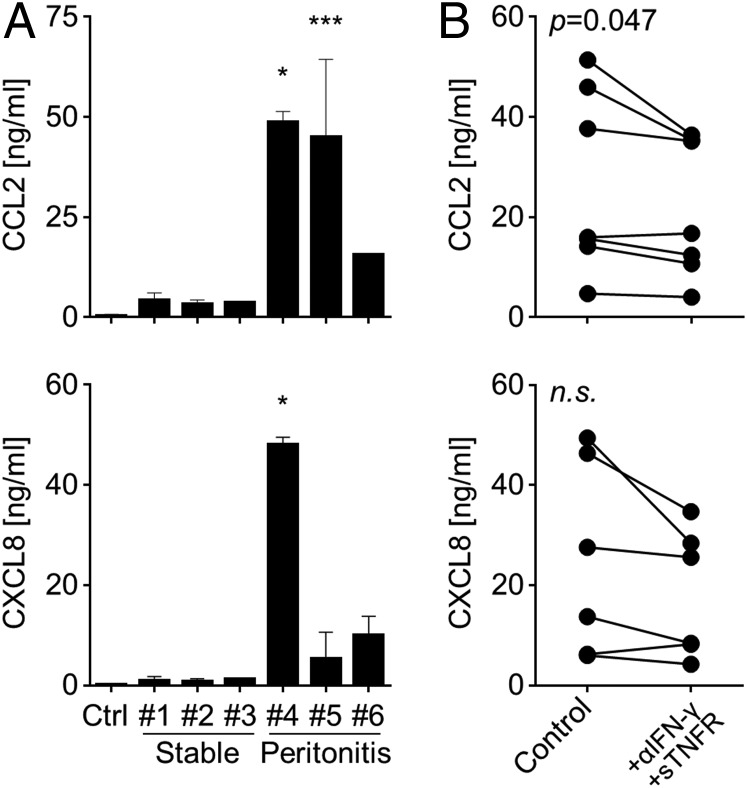
Activation of peritoneal tissue cells by effluent from PD patients with acute peritonitis. (**A**) Growth-arrested peritoneal mesothelial cells from human omentum (*n* = 2–4) were exposed to peritoneal effluent obtained from three stable PD patients in the absence of any inflammation (#1–3) and from three patients presenting with acute peritonitis (#4: *Enterobacter sp.*, #5: *E. coli*, and #6: *Acinetobacter sp.*). Data shown are levels of CCL2 and CXCL8 secreted into the culture medium over 24 h by ELISA (median ± interquartile range). Data were analyzed using Kruskal–Wallis tests combined with Dunn’s multiple comparisons tests. Asterisks indicate significant differences compared with medium controls (Ctrl). (**B**) Mesothelial cells were exposed to peritoneal effluent from patients presenting with peritonitis, in the absence or presence of 10 μg/ml sTNFR and 10 μg/ml anti–IFN-γ. Data shown are expressed as percent inhibition of CCL2 and CXCL8 secretion over 24 h, compared with untreated controls. Data were analyzed using Wilcoxon matched–pairs signed rank tests. Each data point represents an independent experiment.

We next sought to address the physiological relevance of the findings above in more detail. Our previous work already demonstrated that peritoneal effluent of patients presenting with acute peritonitis can contain considerable levels of TNF-α and IFN-γ (13–15). We therefore tested whether cytokines released into the dialysis fluid during acute infection had similar bioactivity to unconventional T cell–conditioned media and the recombinant proteins themselves. In this study, the effluent from three patients with peritonitis induced CCL2 and CXCL8 secretion by mesothelial cells while effluent from stable patients showed only background activity (Fig. 7A). This chemokine production was in part blocked by combined pretreatment of the infected effluent with sTNFR and anti–IFN-γ (Fig. 7B). These experiments demonstrate that TNF-α and IFN-γ are produced locally in response to bacterial pathogens at concentrations that are sufficiently high to affect the cells lining the peritoneal cavity, and that targeting cytokine production by unconventional T cells may diminish local inflammation.

### Clinical outcome from peritonitis depends on the capacity of the causative pathogen to produce unconventional T cell ligands

**FIGURE 8.**
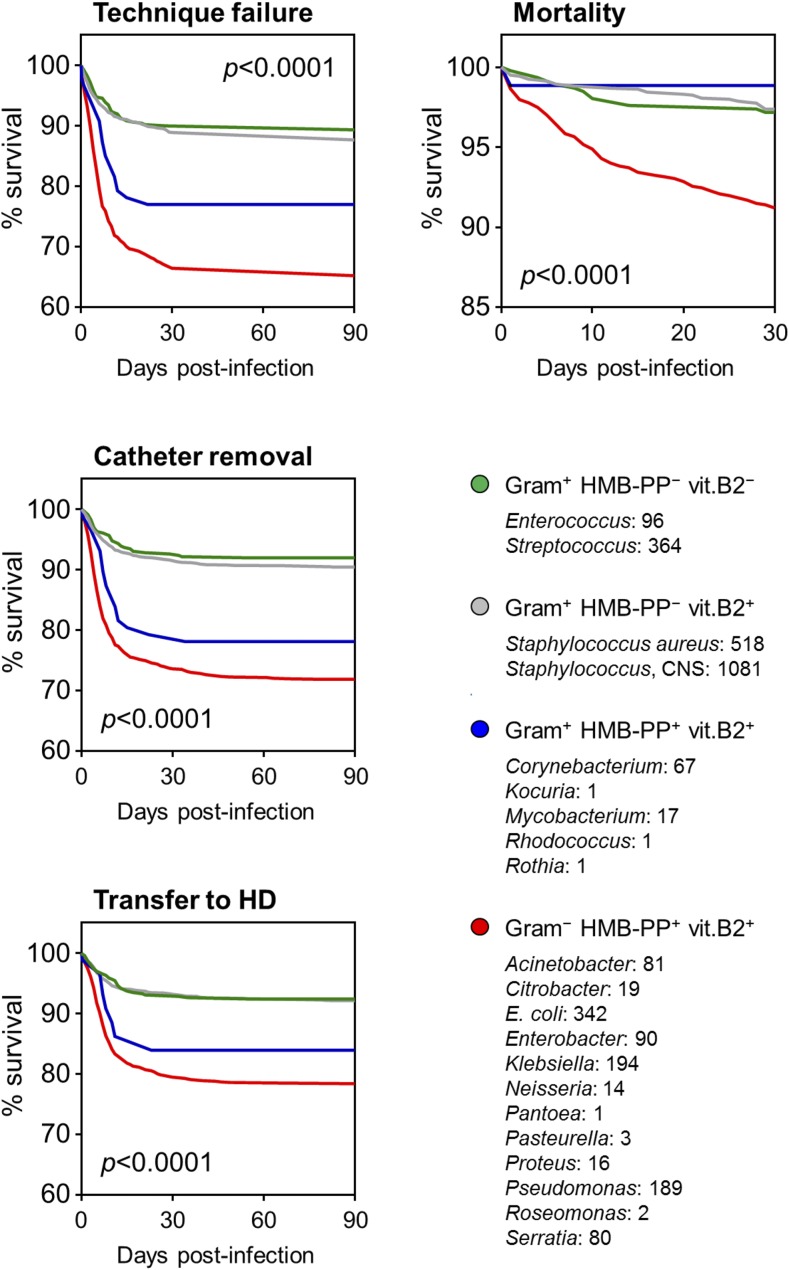
Association of first-time peritonitis caused by HMB-PP^+^ and vit.B2^+^ bacteria with poor clinical outcome. Cumulative rates of technique failure (top left), mortality (top right), catheter removal (middle), and transfer to permanent HD (bottom) of patients from the ANZDATA registry with first-time peritonitis, grouped into infections with Gram^+^HMB-PP^−^vit.B2^−^ (green), Gram^+^HMB-PP^−^vit.B2^+^ (gray), Gram^+^HMB-PP^+^vit.B2^+^ (blue), or Gram^−^HMB-PP^+^ vit.B2^+^ bacteria (red); episodes caused by Gram^−^HMB-PP^−^ (e.g., *Legionella spp.*) or Gram^+^HMB-PP^+^vit.B2^−^ species (e.g., *L. monocytogenes*) were not recorded and/or were too rare for this comparison. Numbers indicate the number of cases of acute peritonitis caused by the listed organisms. Comparisons were made using log-rank tests.

Acute inflammatory events make a major contribution to tissue fibrosis. In particular, peritonitis has a direct effect on peritoneal membrane morphology and function (21, 22) and is hence a major reason for technique failure in PD patients. Yet, little is known about the role of unconventional T cells in these processes. Given the contribution of Vγ9/Vδ2 T cells and MAIT cells to the local immune response to HMB-PP^+^ and vit.B2^+^ bacteria, respectively, we investigated the short- and midterm impact of infections by such organisms on the clinical outcome in 5071 PD patients presenting with first-time peritonitis recorded in the Australia and New Zealand Dialysis and Transplant (ANZDATA) Registry (Table I). In accordance with our earlier findings in a much smaller subgroup of the same cohort (14), infections by HMB-PP^+^ bacteria were associated with poorer outcomes as determined by higher rates of catheter removal, permanent transfer to HD, and overall technique failure, compared with HMB-PP^−^ infections (Fig. 8, Table II). This was true for episodes of peritonitis caused by both Gram^+^ and Gram^−^ species, thereby identifying the utilization of the nonmevalonate pathway of isoprenoid biosynthesis by the causative organism as useful predictive marker and implying that Vγ9/Vδ2 T cell–driven responses may contribute to overall clinical outcome. Within the HMB-PP^+^ group, Gram^−^ organisms caused even more severe complications than Gram^+^ species, including significant mortality within the first mo after the onset of acute peritonitis.

**Table I.** Characteristics of all PD patients analyzed in the current study

	Cardiff	ANZDATA
No. of stable patients	45	n/a
Age (mean ± SD)	69.1 ± 13.5	n/a
Women (%)	18.6	n/a
d on PD (mean ± SD)	624 ± 546	n/a
No. of patients presenting with acute peritonitis	101	5,071
Age (mean ± SD)	66.0 ± 13.3	60.1 ± 16.9
Women (%)	32.6	43.9
d on PD (mean ± SD)	936 ± 856	1010 ± 791
No of culture samples obtained (%)	0.0	0.4
Culture negative episodes (%)	22.8	16.6
Fungi (%)	1.0	2.3
Polymicrobial infections (%)	4.0	6.8
HMB-PP^+^vit.B2^+^ species among single bacteria (%)	34.2	35.3
HMB-PP^−^vit.B2^+^ species among single bacteria (%)	42.5	50.2
HMB-PP^−^vit.B2^−^ species among single bacteria (%)	23.3	14.5

n/a, not applicable.

**Table II.** Risk of technique failure in PD patients presenting with first-time peritonitis, depending on the causative pathogen

ANZDATA (*n* = 5071)	Overall Technique Failure (90th d)		Mortality (30th d)		Catheter Removal (90th d)		Transfer to Interim HD for at Least 30 d		Transfer to Permanent HD (90th d)	
	OR (95% CI)	*p*	OR (95% CI)	*p*	OR (95% CI)	*p*	OR (95% CI)	*p*	OR (95% CI)	*p*
Reference: culture-negative	1.0		1.0		1.0		1.0		1.0	
Gram^+^HMB-PP^+^vitamin B2^+^	2.4 (1.389–4.129)	0.002	0.262 (0.043–2.360)	0.262	3.2 (1.812–5.637)	***	7.3 (1.606–33.143)	0.010	2.3 (1.253–4.398)	0.008
Gram^+^HMB-PP^−^vitamin B2^+^	1.1 (0.866–1.468)	0.374	0.737 (0.455–1.192)	0.213	1.2 (0.895–1.637)	0.215	3.2 (1.125–9.353)	0.029	1.0 (0.763–1.437)	0.775
Gram^+^HMB-PP^−^vitamin B2^−^	0.9 (0.662–1.381)	0.812	0.794 (0.409–1.543)	0.497	1.0 (0.658–1.523)	0.998	2.7 (0.758–9.615)	0.126	1.0 (0.655–1.551)	0.971
Gram^−^HMB-PP^+^vitamin B2^+^	4.3 (3.318–5.500)	***	2.6 (1.713–4.037)	***	4.4 (3.358–5.946)	***	9.0 (3.242–25.319)	***	3.4 (2.506–4.550)	***
Reference: culture-negative	1.0		1.0		1.0		1.0		1.0	
HMB-PP^+^	4.1 (3.194–5.271)	***	2.43 (1.589–3.737)	***	4.3 (3.285–5.792)	***	8.9 (3.201–24.863)	***	3.3 (2.449–4.425)	***
HMB-PP^−^	1.0 (0.843–1.405)	0.515	0.75 (0.474–1.184)	0.217	1.2 (0.868–1.558)	0.312	3.1 (1.099–8.873)	0.033	1.0 (0.766–1.408)	0.808
Reference: culture-negative	1.0		1.0		1.0		1.0		1.0	
Vitamin B2^+^	2.1 (1.703–2.734)	***	1.4 (0.939–2.129)	0.097	2.3 (1.789–3.074)	***	5.5 (2.021–15.235)	0.001	2.0 (1.403–3.049)	***
Vitamin B2^−^	0.9 (0.662–1.381)	0.812	0.8 (0.409–1.543)	0.497	1.0 (0.658–1.523)	0.998	2.7 (0.758–9.615)	0.126	0.9 (0.493–1.689)	0.771
Reference: HMB-PP^−^	1.0		1.0		1.0		1.0		1.0	
Gram^+^ HMB-PP^+^	2.2 (1.312–3.689)	0.003	0.424 (0.058–3.098)	0.398	2.7 (1.618–4.670)	***	0.9 (0.700–7.796)	0.167	2.3 (1.248–4.095)	0.007
Gram^−^ HMB-PP^+^	3.9 (3.263–4.721)	***	3.5 (2.488–4.947)	***	3.8 (3.141–4.700)	***	2.9 (1.821–4.623)	***	3.3 (2.614–4.046)	***
Reference: Vitamin B2^−^	1.0		1.0		1.0		1.0		1.0	
Gram^+^ vitamin B2^+^	1.2 (0.892–1.721)	0.201	0.9 (0.480–1.688)	0.742	1.3 (0.896–1.883)	0.167	1.3 (0.526–3.105)	0.588	1.1 (0.748–1.617)	0.629
Gram^−^ vitamin B2^+^	4.5 (3.235–6.168)	***	3.3 (1.832–5.985)	***	4.4 (3.108–6.415)	***	3.4 (1.420–7.933)	0.006	3.3 (2.302–4.874)	***
Reference: Gram^−^HMB-PP^+^vitamin B2^+^	1.0		1.0		1.0		1.0		1.0	
Gram^+^ HMB-PP^+^vitamin B2^+^	0.6 (0.335–0.939)	0.028	0.12 (0.017–0.877)	0.037	0.7 (0.423–1.211)	0.212	0.805 (0.245–2.648)	0.721	0.228 (0.385–1.255)	0.695
Gram^+^ HMB-PP^−^vitamin B2^+^	0.3 (0.217–0.321)	***	0.3 (0.193–0.407)	***	0.3 (0.219–0.336)	***	0.4 (0.218–0.589)	***	0.3 (0.245–0.392)	***
Gram^+^ HMB-PP^−^vitamin B2^−^	0.2 (0.162–0.309)	***	0.3 (0.167–0.546)	***	0.2 (0.156–0.322)	***	0.3 (0.126–0.704)	0.006	0.3 (0.205–0.434)	***

Episodes were defined based on the microbiological culture results; bacteria species identified were grouped according to Gram status and the presence or absence of HMB-PP and/or vitamin B_2_ in their metabolism. Odds ratios (OR) were determined using binary logistic regression, in comparison with the indicated reference groups. Clinical outcomes examined were catheter removal, transfer to permanent HD, transfer to interim HD and death, as well as overall technique failure. CI, confidence interval.

***p < 0.001.

Because all HMB-PP^+^ bacteria in the ANZDATA cohort were also positive for vitamin B_2_ (and did not include *L. monocytogenes* as the only relevant HMB-PP^+^vit.B2^−^ pathogen in PD patients), outcome predictions based on the presence of the vitamin B_2_ pathway followed closely those seen for HMB-PP^+^ organisms (Table II). However, differential analysis of HMB-PP^−^ infections allowed us to determine the clinical impact of vit.B2^+^ species in that patient subgroup. In this study, vit.B2^+^ infections showed a trend toward higher rates of catheter removal, compared with vit.B2^−^ infections, which was also reflected in total technique failure rates (Fig. 8). However, no such differences between vit.B2^+^ and vit.B2^−^ infections were seen with regard to transfer to HD or mortality. This relatively benign course of HMB-PP^−^vit.B2^+^ peritonitis may have been due to the high prevalence of infections caused by the comparatively avirulent skin commensal *Staphylococcus epidermidis* and related coagulase-negative species (67.6% of all infections in this group). As expected (23, 24), *S. aureus* infections were associated with a considerably greater risk of technique failure and catheter removal than coagulase-negative staphylococcal infections (data not shown). These findings indicate that although the presence of the vitamin B_2_ pathway alone may not be sufficiently predictive of clinical outcome in that patient group, MAIT cells are nevertheless likely to make a contribution to the overall inflammatory response during acute peritonitis caused by vit.B2^+^ organisms.

### Unconventional T cell–driven epithelial–mesenchymal transition of peritoneal mesothelial cells

**FIGURE 9.**
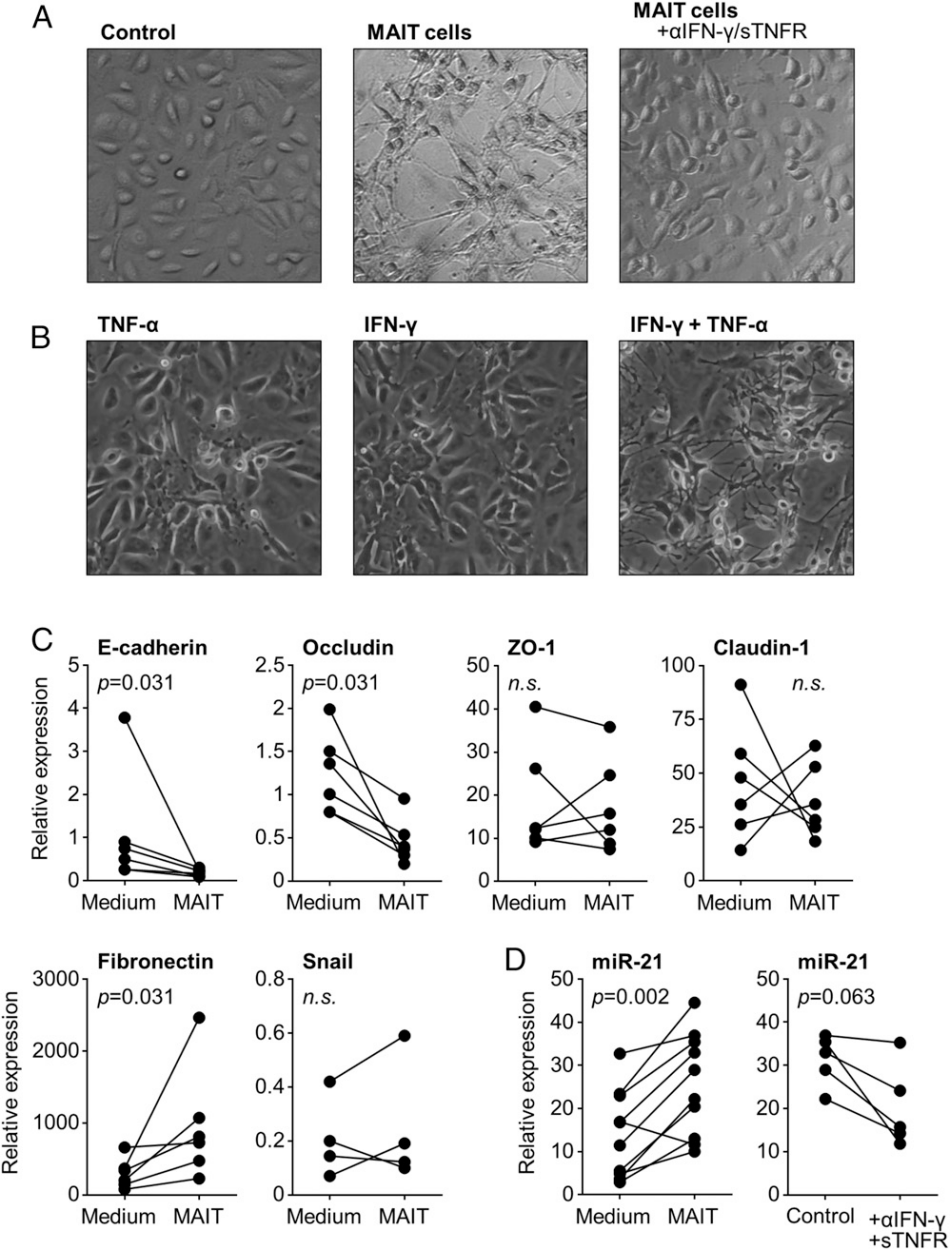
Unconventional T cell–induced reprogramming of peritoneal mesothelial cells. Growth-arrested peritoneal mesothelial cells from human omentum were cultured in medium alone or exposed to supernatants derived from activated MAIT cells, in the absence or presence of 10 μg/ml sTNFR and 10 μg/ml anti–IFN-γ (**A**), or stimulated with 5 ng/ml TNF-α and IFN-γ, alone or in combination (**B**). Images were captured after 24 h in culture with a light microscope at original magnification ×20 and are representative of three to four individual donors. (**C**) Expression of epithelial (E-cadherin, occludin, zona occludens-1 [ZO-1], and claudin-1) and mesenchymal markers (fibronectin and Snail) by mesothelial cells after 24 h exposure to MAIT cell supernatants, as determined by quantitative PCR as relative expression compared with 1000 copies of GAPDH as housekeeping gene. (**D**) Expression of miR-21 by mesothelial cells after 24 h exposure to MAIT cell supernatants in the absence or presence of 10 μg/ml sTNFR and 10 μg/ml anti–IFN-γ, as determined by quantitative PCR as relative expression compared with miR-191 as reference miR. Data were analyzed using Wilcoxon matched–pairs signed rank tests or paired *t* tests. Each data point represents an individual patient.

Finally, we investigated the functional impact of activated unconventional T cells on the surrounding tissue. Inflammatory mediators including TNF-α have previously been associated with the induction of an epithelial–mesenchymal transition (EMT)–like process in mesothelial cells (25), and IFN-γ has been identified as major driver of tissue fibrosis in the peritoneal cavity (22). In this study, primary omentum-derived mesothelial cells exposed to supernatants from activated Vγ9/Vδ2 T cells (data not shown) and MAIT cells (Fig. 9A) underwent striking changes from an epithelial-like appearance to a spindled fibroblastic shape within 24 h. Such pronounced morphological changes were greatly diminished when neutralizing TNF-α and/or IFN-γ in the unconventional T cell–conditioned media prior to addition to mesothelial cells (Fig. 9A). As controls, similar effects were observed when mesothelial cells were cultured in the presence of TNF-α or IFN-γ, particularly when both cytokines were combined (Fig. 9B). In agreement with these morphological changes, the expression of epithelial cell–associated markers such as E-cadherin and occludin by mesothelial cells was downregulated upon exposure to MAIT cell–conditioned media, whereas the mesenchymal marker fibronectin was upregulated, as determined by RT-quantitative PCR (Fig. 9C). A similar upregulation of fibronectin expression was seen with Vγ9/Vδ2 T cell–conditioned medium, whereas effects on E-cadherin and occludin were less pronounced, most likely because of the generally lower levels of TNF-α and IFN-γ in the preparations used for those experiments, compared with MAIT cell–conditioned medium (data not shown). As viability control, expression of IL-6 was greatly enhanced by unconventional T cells at the mRNA level (data not shown), in agreement with the protein data. In addition to fibronectin, MAIT cell–stimulated mesothelial cells also expressed elevated levels of the miR miR-21, which has been linked to TGF-β induced EMT in mesothelial cells and to membrane fibrosis in patients receiving PD (M. Lopez-Anton and D.J. Fraser, unpublished observations). This miR-21 induction by MAIT cells was decreased upon neutralization of TNF-α and IFN-γ (Fig. 9D). Further epithelial (zona occludens-1; claudin) and mesenchymal markers (Snail, collagen-1, and α-smooth muscle actin) were not significantly affected by unconventional T cell–secreted factors (Fig. 9C, data not shown), indicating incomplete initiation of the EMT process under these conditions. Notably, although mesothelial cells treated with a combination of TNF-α and IFN-γ downregulated the expression of all epithelial markers tested (data not shown), only Vγ9/Vδ2 T cells and MAIT cells triggered a parallel upregulation of the mesenchymal marker fibronectin. These data imply that unconventional T cells may produce as yet unidentified mediators, in addition to TNF-α and IFN-γ, that may affect mesothelial cells and reprogram their morphology and phenotype. Taken together, these findings indicate that microbe-responsive unconventional T cells have the capacity to trigger local tissue remodeling with potential consequences for peritoneal membrane integrity and long-term clinical outcome.

## Discussion

By combining cross-sectional and longitudinal sampling in PD patients together with a functional characterization of peritoneal immune and tissue cells ex vivo and an epidemiological analysis of organism-dependent outcomes, we show that Vγ9/Vδ2 T cells and MAIT cells specifically accumulate locally during infections with bacteria producing HMB-PP and vitamin B_2_ derivatives, respectively, and that utilization of the HMB-PP and vitamin B_2_ pathways by the causative organism represents an effective predictive marker for technique failure. To our knowledge, our data thus provide the first conclusive evidence in humans for a ligand-specific role of Vγ9/Vδ2 T cells and MAIT cells at the site of infection. Because of the limited access to relevant patient samples, such a physiological role has so far only been hinted at in complex experimental animal models (26–28). Our data also show that such unconventional human T cells are major producers of IFN-γ and TNF-α in response to microbial pathogens, thereby affecting the cells lining the peritoneal cavity and amplifying local inflammation. These findings are likely to be applicable to other acute infections where local activation of unconventional T cells contributes to the antimicrobial inflammatory response.

The present study demonstrates that PD patients offer exceptional opportunities for immunological studies into acute disease. First, peritoneal effluent can be sampled repeatedly and noninvasively, thus providing continuous access to the site of infection on the first d of microbial infection, before antibiotic therapy commences, to study the development and resolution of acute responses. Second, stable PD patients without overt inflammation can serve as age- and gender-matched noninfected controls in cross-sectional studies. Third, peritonitis remains a relatively frequent complication of PD therapy, with typical incidences in the U.K. of one episode per 15–30 patient mo (29). Thorough immunological profiling of a stable PD cohort therefore allows to establish preinfection baseline parameters in individuals prone to develop peritonitis later on. Fourth, PD-related peritonitis can be caused by Gram-positive and Gram-negative bacteria as well as fungi, thereby allowing to study the local immune response to a wide spectrum of pathogens under closely related conditions. Because acute infection and associated inflammation remain a major cause of treatment failure and even death in PD patients (30), our findings not only improve our insight into the complex local cell interactions in early infection but also provide relevant clues to the mechanisms that underpin the clinical severity of infectious episodes and are readily translatable to improve patient management and outcomes.

Many unconventional T cell populations are rare in humans, with NKT cells typically representing 0.01–0.5% of T cells in blood and frequencies of germline-encoded mycolyl–reactive T cells being even lower (31, 32). Given the rare nature of these populations and the low prevalence of organisms that produce the corresponding ligands in PD patients, we focused on Vγ9/Vδ2 T cells and MAIT cells, the two most abundant unconventional T cell populations in the peritoneal cavity. Our data provide in vitro and in vivo evidence for the specificity of peritoneal Vγ9/Vδ2 T cells and MAIT cells for the corresponding ligands, HMB-PP (33) and vitamin B_2_ derivatives (16, 34). In particular, our research suggests that Vγ9/Vδ2 T cells and MAIT cells discriminate locally between different organisms and accumulate rapidly at the site of infection, where they engage local monocytes and neutrophils as well as tissue cells, and orchestrate early inflammatory events (10, 13, 14). Although others have suggested that circulating MAIT cells decrease in certain clinical scenarios sufficiently to reach statistical significance (35), to our knowledge, our data indicate for the first time that local recruitment of MAIT cells indeed takes place in acute infection. Unconventional T cell–driven responses are likely to contribute to the transition from local innate immunity to Ag-specific adaptive immunity (11) and have potential to be exploited for improved patient management through novel diagnostics, therapeutics and vaccines. As such, we show in this study that local Vγ9/Vδ2 T cells and MAIT cells represent dominant sources of IFN-γ and TNF-α in response to diverse bacteria. As these cytokines control leukocyte recruitment to the site of infection but also possess profibrotic functions, unconventional T cells represent key regulators of acute immune responses as well as collateral tissue damage, ultimately affecting outcomes.

The present study identified unconventional T cell–derived TNF-α and IFN-γ as potent stimulators of primary mesothelial cells and peritoneal fibroblasts, the two major types of tissue cells constituting the peritoneal membrane. Through induction of IL-6 as well as chemokines such as CCL2, CXCL8, and CXCL10, Vγ9/Vδ2 T cells and MAIT cells are likely to enhance local inflammation and contribute to further recruitment of monocytes, neutrophils, and lymphocytes to the site of infection. This interaction with local tissue cells complements our earlier findings on a potent cross-talk of Vγ9/Vδ2 T cells and MAIT cells with monocytes and neutrophils triggering chemokine production and leading to monocyte and neutrophil survival and acquisition of APC features (10, 11, 13, 14). Taken together, these findings indicate that Vγ9/Vδ2 T cells and MAIT cells play a crucial role in driving local inflammatory events by engaging both immune and nonimmune cells at the site of infection by organisms producing the corresponding ligands. The timely detection of Vγ9/Vδ2 T cell and MAIT cell responses in PD patients presenting with acute peritonitis may therefore allow an “immune fingerprint”–based point-of-care definition of the causative pathogen, which would improve early patient management by targeting treatments more efficiently than current empirical approaches, reducing unnecessary exposure to broad-spectrum antibiotics, and identifying individuals at increased risk of subsequent complications who may require prolonged hospitalization (15, 36).

The orchestration of early antimicrobial responses by unconventional T cells is likely to contribute to pathogen clearance and wound healing and thus be beneficial to the host in different infectious scenarios. However, the situation is different in individuals receiving PD who are highly susceptible to inflammation-related tissue damage with immediate consequences for their treatment (37–39). In particular, IL-6 has been linked to tissue fibrosis via induction of Th1 cell responses as a consequence of peritoneal inflammation (22). Unconventional T cells are therefore likely to contribute to scarring in the peritoneal cavity, both directly via the local release of IFN-γ and indirectly via induction of IL-6 production by mesothelial cells and fibroblasts. Our present study identified striking morphological and phenotypical changes of mesothelial cells in response to Vγ9/Vδ2 T cells and MAIT cell secreted cytokines, changes that are linked to infection-driven peritoneal fibrosis and technique failure. Such local tissue remodeling by downregulating epithelial features and inducing expression of mesenchymal markers will ultimately affect the integrity of the peritoneal membrane and affect long-term clinical outcomes. Together, these complex interactions may, at least in part, explain why organisms that are capable of activating local unconventional T cells are frequently associated with higher rates of technique failure in PD patients. Besides predicting important clinical outcomes, unconventional T cell–driven responses to ligands shared by many microbial pathogens therefore also represent potential targets to suppress overshooting inflammation and limit damage on the peritoneal membrane, with the possibility to deliver treatments locally via the peritoneal catheter. Novel interventions will benefit greatly from the recent elucidation of the BTN3 and MR1 presentation pathways, and the availability of reagents blocking the TCR-mediated recognition of microbial ligands (40, 41), antibiotics shutting off the nonmevalonate and riboflavin biosynthesis (42–45), and biologics interfering with key effector cytokines.
